# Histological, Immunological, and Genetic Analysis of Feline Chronic Gingivostomatitis

**DOI:** 10.3389/fvets.2020.00310

**Published:** 2020-06-03

**Authors:** Natalia Vapniarsky, David L. Simpson, Boaz Arzi, Nopmanee Taechangam, Naomi J. Walker, Carissa Garrity, Evelyn Bulkeley, Dori L. Borjesson

**Affiliations:** ^1^School of Veterinary Medicine, Veterinary Institute for Regenerative Cures, University of California, Davis, Davis, CA, United States; ^2^Department of Pathology, Microbiology and Immunology, School of Veterinary Medicine, University of California, Davis, Davis, CA, United States; ^3^Department of Surgical and Radiological Sciences, School of Veterinary Medicine, University of California, Davis, Davis, CA, United States

**Keywords:** feline oral mucosal disease, immune-mediated oral mucosal inflammation, transcriptome of chronic gingivostomatitis, immunophenotyping of FCGS, chronic feline stomatitis, immunohistochemistry of feline stomatitis

## Abstract

Feline chronic gingivostomatitis (FCGS) is an immune-mediated inflammatory condition affecting the oral mucosa that results in substantial pain and suffering. The goal of this study was to complete an in-depth immunohistochemistry analysis of affected FCGS mucosa, to perform and compare immune cell phenotypes in the blood of FCGS and healthy controls cats, and to determine a transcriptomic profile of the affected and normal oral mucosa of FCGS cats. We hypothesized that cats with FCGS would have circulating activated CD8+ T cells and that tissues would be infiltrated with activated B and T cells with a highly proinflammatory transcriptome. We found that oral mucosal tissues from cats with FCGS have high tissue infiltration of B cells and that T cells include both CD4+ and CD8+ lymphocytes. Cells positive for CD25 (IL2 receptor, indicative of lymphocyte activation) and FOXP3 (indicative of regulatory T cells) were scattered throughout the mucosa. Compared to healthy individuals, cats with FCGS had high circulating CD8+ effector memory cells with a concurrent decrease in central memory cells and evidence of circulating activated CD8+ T cells (CD25+, CD62L−). Gene expression in the affected tissues was enriched for genes associated with T-cell signaling, cell adhesion molecules, leukocyte migration, inflammatory signaling pathways, extracellular matrix-receptor interactions, cytokine-cytokine receptor interactions, and natural killer cell-mediated cytotoxicity, among others. These data are essential to understand disease pathogenesis, to inform mechanism of action studies for future and current therapies, and to help select prognostic biomarkers and potency assays for stem cell treatment of FCGS.

## Introduction

Feline chronic gingivostomatitis (FCGS) is an immune-mediated oral mucosal inflammatory disease of cats with a reported prevalence of 0.7–12% of the cat population in the United States and Europe (1). Immune-mediated diseases result from an imbalance of inflammatory cytokines leading to chronic inflammatory conditions (2). This condition is painful and debilitating and characterized by inflammation of the area lateral to the palatoglossal folds, gingiva, and occasionally the alveolar and the labio-buccal mucosa (1). Ulcerative or ulcero-proliferative lesions are often observed (3).

The etiology of FCGS is elusive and characterized by inappropriate immune response to an unknown antigenic stimulus, although underlying viral and bacterial etiologies have been proposed (4–6). In addition, a recent study demonstrated that cats with FCGS are more likely to live in households with greater than one cat. These data also suggest an underlying infectious etiology (7). Current standard treatments include extraction of premolar and molar teeth or the full dentition with or without supplemental immunosuppressive, pain and antibiotic therapy (1). However, ~30% of cats do not respond to the full mouth tooth extraction, and humane euthanasia, due to poor quality of life, may be elected by the cat's owner (8). Importantly, spontaneous resolution of FCGS has not been reported and curative clinical solutions are in high demand.

Complete blood phenotyping and immunohistochemical and transcriptomic analysis of diseased mucosal tissues in cats with FCGS have yet to be reported. Previous work from our group identified neutrophilia, increased CD8+ T cells and increased proinflammatory cytokines in the blood of cats with FCGS compared to healthy controls (9–11). One study used immunohistochemistry on diseased oral tissues to detect T cells (including CD4 and CD8), B cells and mast cells and found significant increases in the number of these cells within the lamina propria/submucosa of FCGS cats with a predominance of CD8+ cells over CD4+ cells (12). A different study used quantitative PCR on diseased tissues to detect a limited number of proinflammatory genes and toll-like receptors. The authors noted increased mRNA levels of a number of toll-like receptors and pro-inflammatory genes including TNF-α, IFN-γ, IL-1β, and IL-6 (13).

FCGS is a disease with a complex and likely a multifactorial etiology. The aim of this study was to perform a comprehensive evaluation of blood phenotype and oral mucosal immune cell infiltrates and gene expression profiles of healthy and diseased tissues from FCGS cats. We hypothesized that cats with FCGS would have circulating activated CD8+ T cells and that tissues would be infiltrated with activated B and T cells with a highly proinflammatory transcriptome.

## Materials and Methods

### Case Selection

Twenty-three edentulous cats affected by stomatitis (i.e., FCGS cats) were included in this study (7 females and 16 males). FCGS cats ranged from 3 to 14 years of age (median age of 7 years). Cats with FCGS had no other concurrent diseases. In addition, eight healthy age-matched control cats (5 male and 3 female) were included in this study with the age range of 7–14 years (median age of 10 years). Stomatitis index (SDAI) was determined for all cats. Briefly, the cat's owners completed a questionnaire and scored the appetite, activity level, grooming behavior and perceived oral comfort on a scale of 0 to 3. In addition, a board-certified veterinary dentist (BA), scored the severity of oral inflammatory lesions as 0 (no lesion), 1 (mild), 2 (moderate), and 3 (severe). The SDAI score for each cat was calculated (range = 0, no disease, to 20, severe disease). Blood and tissues were obtained with the approval of the animal care and use protocol at University of California (UC) Davis and with an owner-signed informed consent (#19170, Aug. 2016).

### Blood Analyses

A full CBC on blood preserved in EDTA was performed on FCGS (*n* = 23) and healthy (*n* = 8) cats (Bayer ADVIA 120; Bayer Diagnostics, Tarrytown, NY, http://healthcare.bayer.com, Veterinary Medical Teaching Hospital, School of Veterinary Medicine, UC Davis). Routine serum biochemistry was performed for all FCGS cats included in the study. Inclusion into this study was conditioned on no evidence of systemic illness and normal biochemistry panel with exception of hyperproteinemia and hyperglobulinemia (previously reported as common in cats with in FCGS) (10). All cats in this study were confirmed negative for FIV and FeLV. The cats were not tested for feline calicivirus. The mucosal tissues from FCGS cats were collected under general anesthesia from the area lateral to the palatoglossal folds using iris scissors. Full-thickness 6–8 mm biopsies were obtained from grossly affected mucosa and adjacent normal mucosa.

### Histology and Immunohistochemistry (IHC)

Routine histological evaluation was performed on mucosal samples from all 23 FCGS cats. Further histological and IHC analyses were performed on formalin fixed paraffin embedded (FFPE) specimens or fresh frozen tissues from FCGS cats by a board-certified pathologist (NV). Randomly chosen oral mucosa tissue samples from FCGS cats (*n* = 5) were preserved fresh frozen (for detection of CD4 and CD8 antigens) and FFPE (*n* = 5) for detection of the remainder antigens (CD3, CD20, CD25, and FOXP3). All samples were sectioned at 4 μm and processed for routine histology and IHC labeling. In brief, FFPE sections were deparaffinized in xylene and exposed to decreasing concentrations of ethanol followed by water and Tris- buffered saline with Tween. For all IHC sections, endogenous peroxidase activity was blocked by incubation in 0.3% hydrogen peroxide in methanol for 30 min. Antigen retrieval was performed on FFPE samples according to procedures optimized for each antibody (Table 1). Sections were blocked with 1% Fc block (FcR Blocking Reagent, Miltenyi Biotec) and 15% donkey serum in Tween. Sections were incubated with primary antibody (Table 1) overnight at 4°C followed by two consequent washes and application of secondary antibody for 1 h at room temperature. ABC-vectastain kit and NovaRed Peroxidase Substrate Kit (Vector Laboratories) or Diaminobenzidin (DAB) were used for antigen detection in feline samples. The IHC slides were counterstained with Modified Mayer's Hematoxylin (Richard Allan Scientific), then washed in Scott's tap water to blue the stain. Slides were dehydrated and cover slipped using Shandon-Mount mounting medium (Thermo Fisher Scientific). Histological images were viewed and acquired on Olympus BX40 equipped with Olympus DP72 camera and cellSens XV image processing software. All IHC images were captured at 100x and 400x magnifications. The 100x magnification covered the majority of the tissue section, and areas with most intense inflammatory infiltration were positioned in the middle of the capture.

**Table 1:** IHC antibodies and reagents.

	**1-ry Ab**	**2-ry Ab**	**Antigen retrieval**
CD3 (T cells)	Rat anti-feline CD3, clone 3–12, diluted 1/10; Leukocyte Antigen Biology Laboratory, UCD School of Veterinary Medicine, Davis, CA	Anti-rat link; 4+ Detection System (HP604 Biocare Medical, Concord, CA)	Dako Target Retrieval Solution (S1699; Agilent Technologies)
CD4 (T helper cells)	Mouse Clone FE1.7B12, Leukocyte Antigen Biology Laboratory, UC Davis (LABL), 1/10 dilution	Anti-mouse peroxidase (Vectastain ABC kit, Vector Laboratories)	Frozen sections—no antigen retrieval
CD8 (T cytotoxic cells)	Mouse Clone Fe1.10E9, LABL, 1/10 dilution	Anti-mouse peroxidase (Vectastain ABC kit, Vector Laboratories)	Frozen sections—no antigen retrieval
CD25 (T regulatory cells)	Mouse 4C9, 1 μg/ml (1:10 dilution) (Millipore Sigma)	Anti-mouse peroxidase (Vectastain ABC kit, Vector Laboratories)	FFPE IHC, Ag retrieval 1 mM EDTA, 0.05% Tween-20 pH 8.5, steamer 45 min
FOXP3 (T regulatory cells)	Mouse 157B/F4 hybridoma supernatant undiluted (gift of Dr. Alison Banham, University of Oxford, Oxford, UK)	Anti-mouse peroxidase (Vectastain ABC kit, Vector Laboratories)	FFPE IHC, Ag retrieval Citrate buffer, pH 6.0 (Dako)
CD20 (B cells)	Rabbit anti-CD20 (NeoMarker RB-9013-P1; 1/300; Thermo Fisher Scientific)	Anti-rabbit link, 4+ Detection System (GR608, Biocare Medical, Concord, CA)	Dako Target Retrieval Solution (S1699; Agilent Technologies)

### Blood Immunophenotyping

Lymphocyte phenotyping was performed with flow cytometry on either whole blood (CD3, CD4, CD8, and CD21) or on Ficoll isolated peripheral blood mononuclear cells (PBMCs; CD25, FOXP3, CD62L, CD45R). Full lymphocyte phenotyping was performed on randomly selected 12 FCGS and six healthy control cats. For CD3, CD4, CD8, and CD21 immunophenotyping, 100 μL aliquots of whole blood from diseased and healthy patients were prepared and incubated with a primary antibody (either conjugated or unconjugated) for 30 min at room temperature. Blood was subjected to RBC lysis with ammonium chloride buffer for 10 min at room temperature. The cells were then washed twice in flow buffer (DPBS –Ca –Mg + 2% FBS (Atlanta Biologics), 2 mM EDTA, in DPBS). Samples labeled with an unconjugated primary antibody were then incubated with a secondary antibody diluted in 100 μL flow buffer, for 20 min at room temperature. Antibody information is detailed in Table 2. Labeled cells were analyzed on FC500 flow cytometer (Beckman Coulter using Cytomics CXP software (Beckman Coulter) and FlowJo software (Ashland, OR).

**Table 2:** Flow cytometry antibodies, clones, and source.

**Antigen**	**Clone**
CD4 (blood)	Mouse FE1.7B12, unconjugated [Leukocyte Antigen Biology Lab, UC Davis (LABL)], 25 μL
CD8 (blood)	Mouse FE1.10E9, unconjugated (LABL), 25 μL
CD21 (blood)	Mouse CA2.1D6, unconjugated (LABL), 25 μL
CD4 (Ficoll)	Mouse FE1.7B12, biotinylated (LABL), 0.25 μL or 3-4F4 (Southern Biotech), 1 μL
CD8 (Ficoll)	Mouse FE1.10E9 unconjugated (LABL), 1 μL
CD45R (Ficoll)	Mouse RA3-6B2(B220) APC-Cy7 (BioLegend), 1.25 μL
CD25 (Ficoll)	Mouse 9F23-FITC (gift from Dr. Ohno, U. Tokio), 0.2 μL
CD62L (Ficoll)	Mouse LAM1-116-APC (MyBioSource), 0.8 μL
FOXP3 (Ficoll)	Mouse FJK-16Ss-APC (eBioscience), 2.5 μL
Anti-mouse Ig (H+L), F(b)'2	(Jackson Immuno) Donkey α mouse-PE 1:50
Streptavidin-PE	(BD Biosciences) 1:2,000

**Figure 1:**
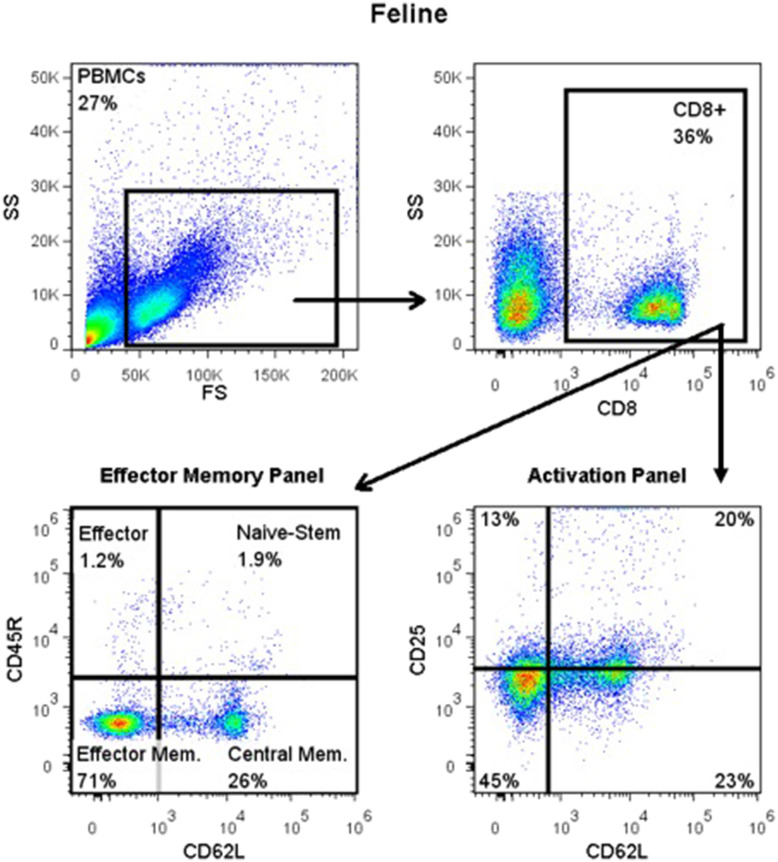
Flow cytometry gating strategy for immunophenotyping of immune cells in the systemic circulation of FCGS (*n* = 12) patients along with healthy (*n* = 6) controls. General lymphocyte population was identified first using forward and side scatter. Next, CD8 immunofluorescent cells were selected out of the lymphocyte gate. Within CD8 gate, percentages of effector memory (CD8+CD45-CD62L−) and central memory (CD8+CD45-CD62L+) cells were interrogated. Similarly, within the CD8 positive population, percentages of activated CD8 cells were quantified (CD8+CD25+CD62L−). CD45R antibody utilized here (also known as CD45RABC) recognizes three (A, B, and C) exons of the CD45 protein. These exons are alternatively spliced to generate up to eight different protein products featuring combinations of zero, one, two, or all three exons. CD45 isoforms show cell-type and differentiation-stage specific expression, a pattern which is quite well-conserved in mammals. These isoforms are often used as markers that identify and distinguish between different types of immune cells. Naive T lymphocytes are typically positive for CD45RA, which includes only the A protein region. Activated and memory T lymphocytes express CD45RO, the shortest CD45 isoform, which lacks all three of the A, B, and C regions. This shortest isoform facilitates T cell activation (15).

For detection of CD45R, CD62L, CD25, and FOXP3 antigens, blood was Ficolled to separate PBMCs, following an established method (14) with some modifications. Briefly, blood was diluted with 2 volumes of warm Tyrodes HEPES buffer (12 mM NaHCO_3_, 138 mM NaCl, 2.9 mM KCl, 10 mM HEPES, 2.5 mM EDTA, pH 7.2). Diluted blood was layered on top of a discontinuous gradient (Ficoll-Paque Plus diluted 6:1 with sterile water, on top of Histopaque-1.119) (Sigma, St. Louis, MO). Samples were centrifuged at 400 g for 20 min with no brake. The PBMC layer was transferred into a fresh tube and washed twice with DPBS before resuspending in flow buffer and labeling cells. Cell surface markers were labeled as detailed above. Samples for intracellular FOXP3 labeling were further processed using a FOXP3 fixation/permeabilization kit (eBioscience), following the manufacturer's instructions. They were then washed twice in flow buffer containing 0.1% saponin (Perm/Wash buffer), and blocked with 2% rat serum in Perm/Wash for 15 min before the FOXP3 antibody was added. Cells were incubated at 4°C for 30 min, then washed twice with Perm/Wash, before suspension in flow buffer. All samples were read on an FC500 cytometer and analyzed with FlowJo software, as described above. The gating strategy is depicted in Figure 1.

### Transcriptome Analysis

Diseased and adjacent healthy mucosal tissues from three, randomly selected FCGS cats were flash-frozen for RNA isolation and transcriptome analysis. Briefly, transcriptome is a study of all genes for which there is RNA transcription that allows determination of which genes are up- or down-regulated in comparison to normal tissue (16). Total RNA was isolated using the RNeasy Mini Kit (Qiagen). The RNA quantity and quality were assessed on a NanoDrop spectrophotometer (Thermo Scientific). RNA library preparation and next generation sequencing were performed according to previously described methods (17). Briefly, 200 ng of total RNA were used for indexing and library preparation which were carried out at the UC Davis Comprehensive Cancer Center's Genomics Shared Resource facility. The libraries were then enriched by high-fidelity PCR amplification (15 cycles) with KAPA HiFi HotStart DNA Polymerase and adapter-specific primers. Subsequently, libraries were combined for multiplex sequencing on an Illumina HiSeq 4,000 System (100-bp, paired-end; ~30 million reads/sample) (18). Expression data was compared between groups (healthy and diseased tissues) and those genes with at least 2-fold expression between healthy and diseased tissue were identified.

### Statistical Analyses

The immunophenotyping data was analyzed with GraphPad Prism version 6.05 software (GraphPad San Diego, CA). The statistical significance between healthy and diseased patients was determined by two-tailed paired *t*-test. A *p*-value < 0.05 was considered statistically significant. Transcriptome analyses data were normalized and analyzed (by DLS) for hierarchical clustering and principle component analysis using JMP (version 14; Cary, NC). Additional analysis was completed using the Database for Annotation, Visualization and Integrated Discovery (DAVID; David.ncifcrf.gov) (19) to assess functional enrichment based on differentially expressed genes between healthy and diseased tissues. Functional KEGG Pathway terms with a false discovery rate < 1 were compared to background values to generate an enrichment score and *p*-values.

## Results

### Clinical Presentation of FCGS

All cats with non-responsive FCGS were edentulous and dental radiographs confirmed the absence of root tips. In all cats, there was severe ulcerative inflammation lateral to the palatoglossal folds and buccal mucosa with some inflammation at the attached gingiva in the area of the premolar-molar teeth. The SDAI scores ranged from 6.25 to 18.27 (mean 18.27). At the time of tissue collection, all cats were receiving pain medication (buprenorphine) and no cats were receiving immunosuppressive therapy, antibiotics or any oral health care products.

### Blood Analysis of FCGS Cats Compared to Healthy

Table 3 summarizes the WBC parameters for cats with FCGS compared to healthy controls. Similar to previous reports, cats with FCGS had a significantly higher WBC count (average 13.41) than healthy controls (9.09; *p* = 0.003) due to increased numbers of neutrophils. Specifically, 39% of the cats presented with a leukocytosis (9/23 cats had a WBC count above the reference interval) due to neutrophilia. Cats with FCGS had normal percentage of CD4+ T cells however the percentage of CD8+ T cells was significantly increased compared to healthy cats (*p* = 0.001) with a resultant decrease in the CD4/CD8 ratio (*p* = 0.01) compared to healthy cats (10). The circulating percentage of CD21+ B cells was also significantly decreased in FCGS cats compared to healthy control cats (*p* = 0.03).

**Table 3:** Differential cell counts in the peripheral blood of FCGS patients compared to healthy controls.

**Parameter**	**Category**	***n***	**Average**	**Patient range**	***P*-value**
WBC number	FCGS	23	13.41	3.9–28.1	**0.003**
	Control	8	9.09	4.4–12.8	
# Lymphocytes	FCGS	23	2.02	0.74–4.87	0.78
	Control	8	2.17	0.67–4.35	
# Neutrophils	FCGS	23	10.14	2.14–22.71	**0.001**
	Control	8	5.87	2.40–8.12	
% CD4+ T cells	FCGS	23	21.9	10.9–35.9	0.24
	Control	8	25.4	15.5–33.1	
% CD8+ T cells	FCGS	23	24.6	10.8–42.7	**0.001**
	Control	8	15.3	8.2–22.2	
CD4/CD8 ratio	FCGS	23	1.00	0.30–2.18	**0.01**
	Control	8	1.77	1.02–3.13	
% CD21+ B cells	FCGS	23	29.1	7.5–14	**0.03**
	Control	8	38.7	1.2–14.9	

*Bold values indicate statistical significance*.

### FCGS Cats Have Profound B and T Cell Tissue Inflammation With Scattered CD25+ Activated Lymphocytes

**Figure 2:**
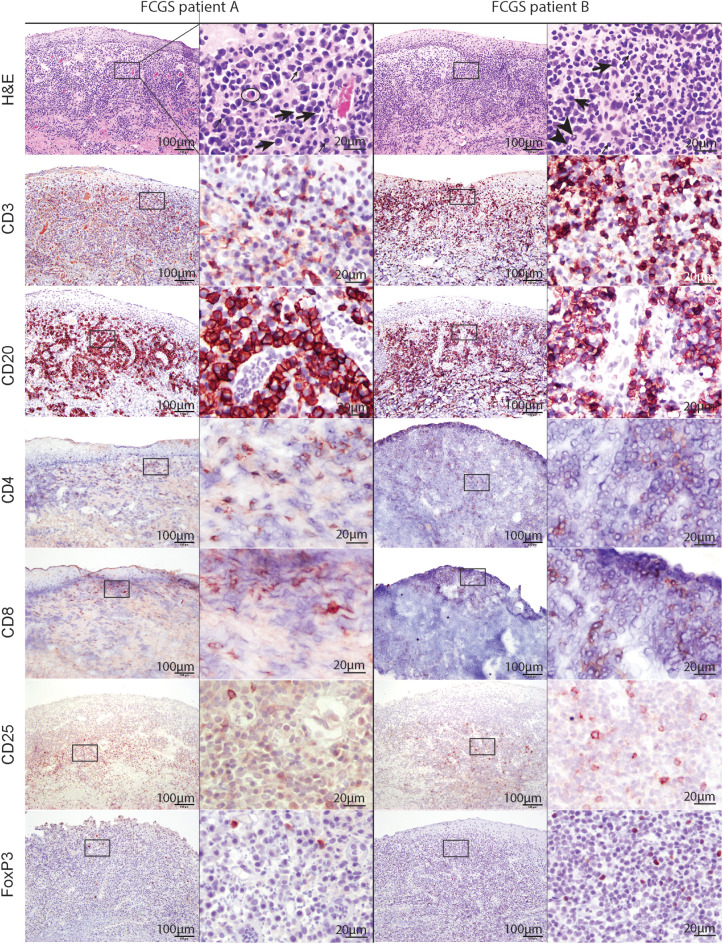
Low (100x) and high (400x) magnification images of hematoxylin and eosin and immunohistochemical sections from two different FCGS patients. The rectangle on low magnification image indicates the area captured on high magnification. Note the abundance of plasma cells (thick arrow) and occasional mott cells (ellipse) in the sections of feline patients relative to a fewer lymphocytes (thin arrow) and neutrophils (arrow head). Mott cell is a plasma cell which cytoplasm is expanded by accumulation of immunoglobulins. Scale bar = 100 μm on low (100x) magnification captures and 20 μm on high (400x) magnification captures.

Histologically, the affected tissues were heavily infiltrated by lymphocytes and plasma cells. According to the suggested histological grading scheme, the severity of lesions ranged from grade 2 to grade 3 (20). In the majority of cases (16/20; 80%), the inflammatory cells were distributed in a band-like pattern encompassing and obscuring the border between epithelium and submucosa (Figure 2, H&E). In the remainder of the cases inflammatory cell infiltration was more scattered or formed indiscrete aggregates. In the majority of cases the mucosal epithelium was hyperplastic with four to 15 keratinocyte layers and prominent rete pegs that extended deep into the submucosa. The submucosal capillaries were congested and lined by plump endothelial cells. There was no evidence of bacteria or viral inclusions or other cytopathology suggestive of viral infection. Lymphocytic infiltrate was dominated by plasma cells with occasional to abundant mott cells (Figure 2, CD3 and CD20). Neutrophils were primarily observed in sections where the mucosa was ulcerated. Occasional neutrophilic aggregates (microabscesses) were observed in the hyperplastic mucosal epithelium adjacent to the ulcerated locations. Mast cells, dendritic cells, and macrophages were occasionally observed and no other cell types such as basophils or eosinophils were present in the examined sections.

T and B cells heavily infiltrated the affected tissues (Figure 2, CD3, and CD20). T cells were present in the superficial mucosa and submucosa (Figure 2, CD3) whereas B cells and mott cells were restricted to the submucosa (Figure 2, CD20).

CD4 and CD8 immunoreactive cells followed the general pattern of T cell distribution (i.e., were primarily located in the mucosa and submucosa). CD25 immunoreactive cells were located in the superficial submucosa and rarely in the mucosa. FOXP3 immunolabeling was primarily nuclear with occasional sections showing cytoplasmic labeling. FOXP3 positive cells were scattered throughout the superficial submucosa, similar to CD25.

### FCGS Cats Have Increased Circulating CD8+ Effector Memory Cells and Activates CD8+ T Cells

**Figure 3:**
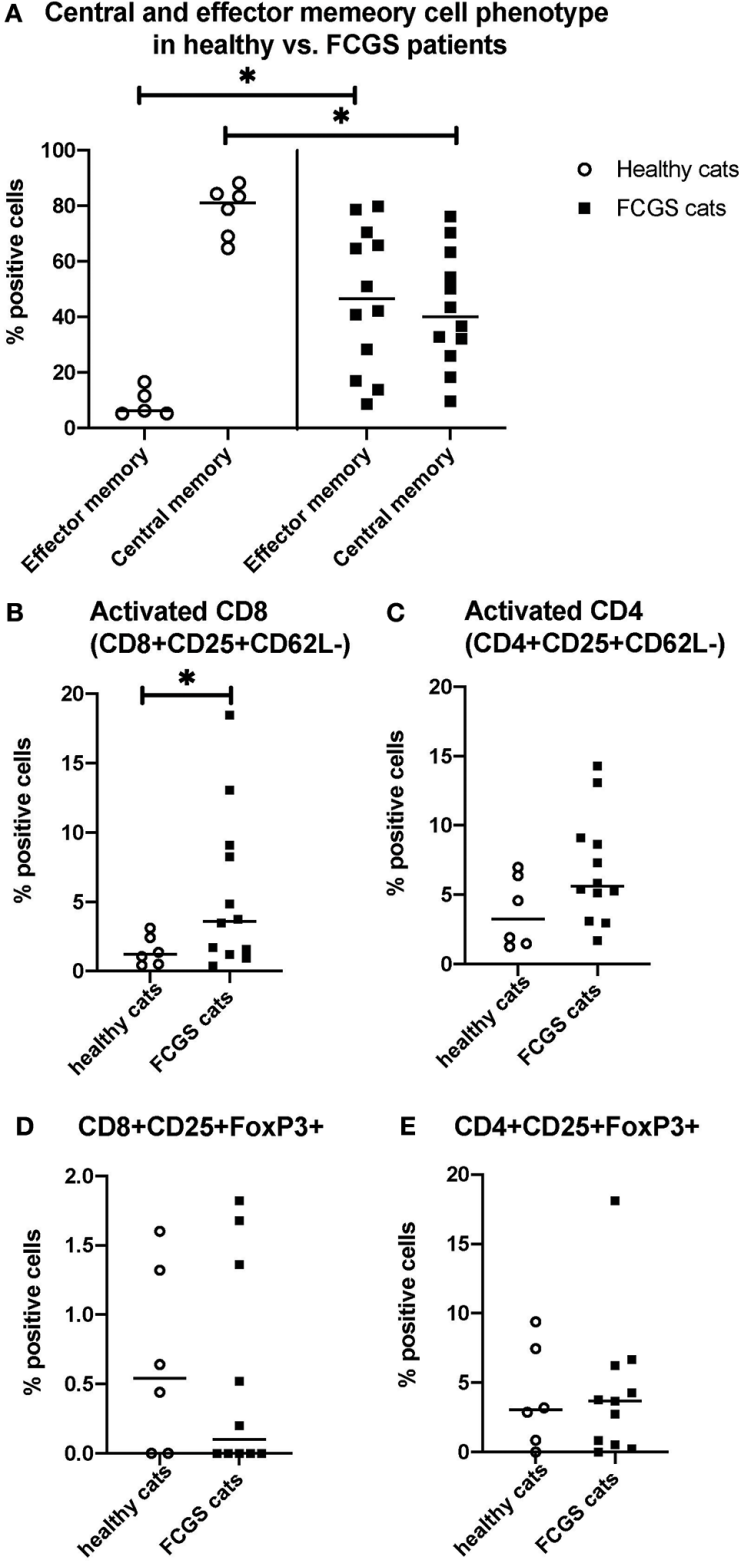
Quantitative assessment of selected immunophenotypes in the circulation of FCGS (*n* = 12) patients compared to healthy controls (*n* = 6), respectively. Significant differences are highlighted by a line with and asterisk. The Y axes reflect the percentage of immunoreactive cells relative to the total lymphocyte population in the sample. **(A)** Percentages of central and effector CD8+ cells in the systemic circulation in healthy and FCGS patients. Percentage of the effector memory (CD8+CD45-CD62L−) lymphocytes is significantly higher in FCGS patients than in healthy controls (*p* < 0.05). Percentages of central memory (CD8+CD45-CD62L+) lymphocytes are significantly lower in FCGS patients than in healthy controls (*p* < 0.05). **(B)** Percentages of activated CD8+ cells are significantly higher in the circulation of FCGS patients than in healthy controls (*p* < 0.05). **(C)** Percentages of activated CD4+ cells are trending higher than in healthy controls. **(D,E)** Percentages of CD25+ FOXP3+ CD8+ and CD25+ FOXP3+ CD4+ lymphocytes, respectively, are trending higher in the FCGS patients.

The most notable and consistent lymphocyte phenotype alteration in FCGS cats was a significant increase in CD8+ effector memory cells (*p* < 0.0001) with an associated reduction in central memory cells (Figure 3A). These CD8+ T cells also had an activated phenotype with increased percentages of CD25+ CD62L- cells (*p* < 0.03; Figure 3B), primarily due to cells that were variably CD25+ but consistently CD62L- (*p* < 0.0001). No significant difference was detected in the activated CD4+ T cell phenotype but the trend was similar to that of activated CD8+ T cells (*p* = 0.066; Figure 3C). There was no difference in FOXP3 expression on CD4 or CD8 positive T cells between healthy and diseased cats. However, 50% of the FCGS cats had notable increases in FOXP3 positive cells (both CD8+ and CD4+, Figures 3D,E). The average CD4+FOXP3 in healthy cats was ~6% FOXP3+ cells. FCGS cats with increased CD4+FOXP3 ranged from 8.3 to 31.8%.

### Oral Mucosal Lesions From FCGS Cats Are Enriched in Genes Associated With T Cell Signaling, Leukocyte Adhesion, Inflammatory Signaling, and NK Mediated Cytotoxicity Among Others

**Figure 4:**
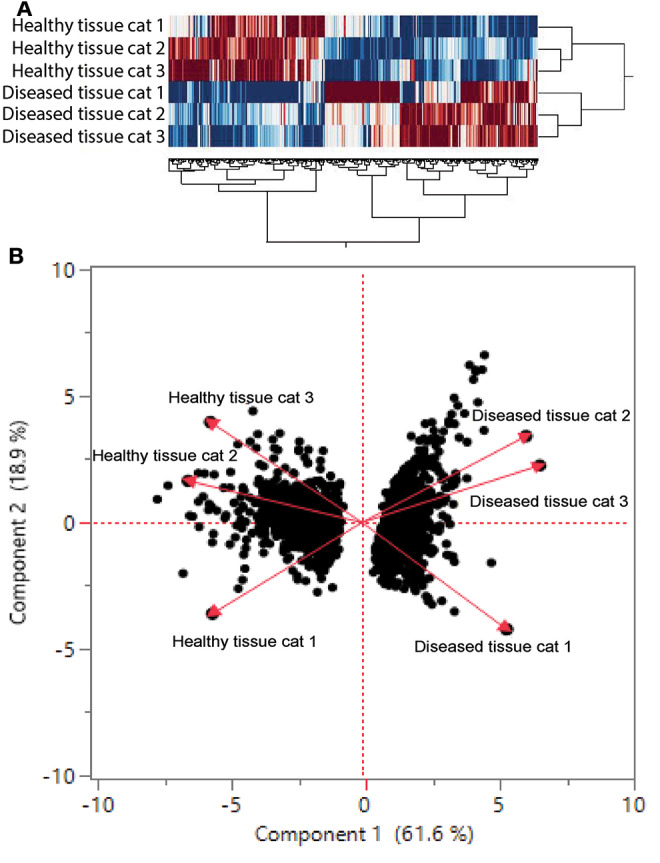
**(A)** Cluster analysis of differentially regulated genes in healthy and diseased tissue from FCGS cats. Note that diseased tissues are clustering differently from healthy. **(B)** Principal component analysis of genes in healthy and diseased cat tissues.

We found 2,310 genes that were differentially regulated at least 2-fold between diseased and healthy cat tissues. Within the differentially expressed genes, 1,331 genes were upregulated, and 979 genes were downregulated. Cluster analysis of the 2,310 differentially expressed genes reveled clear clustering of diseased and healthy tissues (Figure 4A). Principle component analysis revealed similar clustering of genes on components of disease state such that genes from diseased tissues clearly clustered together (Figure 4B). DAVID's platform analysis of upregulated genes (diseased vs. healthy tissues) revealed 1.8–4.7-fold enrichment of genes associated with T-cell signaling, cell adhesion molecules, leukocyte trans-endothelial migration, NF-kappa B signaling pathways, extracellular matrix-receptor interactions, cytokine-cytokine receptor interactions, complement and coagulation cascades, Fc-gamma mediated phagocytosis, and natural killer cell mediated cytotoxicity, among others pathways (Supplemental Table 1). Conversely, DAVID's platform analysis of downregulated genes revealed 1.5–3.1-fold enrichment of genes associated with Axon guidance, Metabolic pathways, Rap1 signaling pathway, Hippo signaling pathway and tight junctions (Supplemental Table 1).

## Discussion

This study is the first to evaluate the histological, immunological, and genetic aspects of FCGS in a comprehensive and combined fashion. We found that CD8+ lymphocytes are dysregulated in FCGS. In addition, we demonstrated that there is a significant increase in effector memory CD8 lymphocytes and a corresponding significant decrease in central memory CD8 lymphocytes in FCGS affected cats as compared to healthy controls. Furthermore, there was a concurrent systemic increase of activated CD8 lymphocytes in FCGS affected cats as compared to healthy controls. In oral mucosal tissues, there was a striking difference in gene expression between FCGS lesions and healthy mucosal tissue. Primarily, the genes from diseased tissues clustered together and were significantly different from genes expressed in healthy oral mucosa. The immunohistochemical assessment of FCGS tissues consistently reveled predominance of B-cells which is in contrast to a decrease in B-cells in the circulation. In concert, these findings provide further insights into the FCGS pathogenesis and provide directions for development of targeted treatments to achieve cure.

In general, T cells can be divided into two major subgroups that are characterized by the expression of CD4 or CD8. The primary function of CD4+ cells is in helping to regulate other immune cells through the release of cytokines or by direct cell contact. The subsets of CD4+ helper T cells include T helpers 1, 2, and 17 (Th1, Th2, and Th17, respectively), follicular T cells (TFH), and regulatory T cells (T reg). CD8+ cells are cytotoxic T cells that typically kill target cells infected with viruses, intracellular bacteria, or cells that have undergone neoplastic transformation. Following encounter with the cognate antigen (viral, bacterial, or altered cellular) CD8+ cells undergo clonal expansion and concomitant differentiation into an effector phenotype that is characterized by production of abundant pro-inflammatory cytokines and the ability to kill target cells. A small fraction of these cells acquires a memory phenotype. The memory phenotype confers long-term antigen-independent survival to CD8+ T cells (21). Within this cohort of memory T cells some are equipped with the ability to enter secondary lymphoid tissue (central memory), such as a lymph node or spleen, while others (effector memory) are able to attach to the endothelium and “home” to the sites of inflammation (22) in other organs such as skin, liver, and oral mucosa.

Our histological and immunohistochemical investigation revealed a striking abundance of B cells in the inflammatory infiltrate. Interestingly, the abundance of B cells in the affected mucosal tissues coincided with a decrease in the number of circulating B cells systemically. This altered distribution of B cells in FCGS cats may be explained by mobilization of B cells to the secondary lymphoid organs such as lymph nodes through the influence of proinflammatory cytokines such as IFN gamma and TNF alfa (23). Indeed, lymphadenopathy consistent with lymph node reactivity is a common finding in this disease (1). In FCGS histological specimens, CD4+ and CD8+ immunolabeling revealed approximately equal ratio of these cells in the lamina propria. Our study population consisted of edentulous FCGS patients with chronic inflammation histologically graded as two or three (i.e., cats that did not respond to extraction therapy). The choice of this population stemmed from the knowledge that FCGS affected cats that still have teeth (i.e., before extraction therapy) typically have concurrent moderate to severe periodontitis (24). The presence of both diseases concomitantly could potentially bias and complicate the interpretation of the results. By selecting edentulous cats with FCGS, we narrowed our focus on more “pure” disease. Immunohistochemical analyses were performed on five randomly selected sections with adequate representation of mucosa and submucosa. It is possible that this limited sample size was not fully representative of the disease. One seminal study performed a similar histopathological investigation in FCGS cats (12). The authors reported higher numbers of CD8+ lymphocytes than CD4+ lymphocytes and did not find a predominance of B cells in the histological sections. It is difficult to compare our results to the results reported by Harley at al. because the status of the cat's dentition in the study population was not provided (12).

In FCGS cats, the CD4/CD8 ratio in the peripheral blood was dysregulated and was much lower than in healthy controls (1.00 vs. 1.77, respectively). This is in agreement with our previous studies that demonstrated that FCGS affected cats had a skewed peripheral blood CD4/CD8 ratio due to increased circulating CD8+ cells (9, 10). Specifically, the CD4/CD8 ratio provides a marker of immune activation and immune senescence. In that context, CD4/CD8 ratio might represent a good predictor of host-viral events and a window into the host immune response capabilities (25). It would also be essential to perform a similar evaluation in a cohort of non-edentulous cats with FCGS as it is a more 'virgin' and complex form of the disease. Such a comparison may offer additional information on the pathogenesis of this condition.

We demonstrated, for the first time, a systemic dysregulation of CD8 effector memory and central memory cells in cats with FCGS (Figure 3). These findings suggest unresolved inflammation in which CD8+ T-cells are activated more than once and remain in an activated state. The concurrent reduction in central memory cells supports this concept because central memory cells become effector memory cells when activated more than once by the same antigen. The effector memory T-lymphocytes do not express L-selectin, as they circulate in the periphery and have immediate effector functions upon encountering antigen (26, 27). The predominance of CD8+ T cells in blood and tissue and the shift in these cells to activated effector memory cells is in line with the proposed underlying viral etiology of FCGS, with feline calicivirus being one candidate (4, 28). The definitive role of this virus however could not be established in experimentally induced infections (29). Thus, the search for other pathogens as contributors of antigenic stimuli should continue, particularly in light of transcriptomic data (discussed below). Further exploration of CD8+ T-cell phenotypes, and their functional potency, is warranted in part because normalization of CD8+ T cell numbers was demonstrated to coincide with clinical recovery upon treatment with MSCs (10).

Cells immunoreactive for CD25 and FOXP3 were abundant in diseased tissues of FCGS cats but there were no significant differences in quantity of these lymphocyte subsets in the circulation (Figure 3). The limitation of this study is that CD25 and FOXP3 immunolabeling does not specify if these are CD4+ or CD8+ lymphocyte subsets. Multicolor immunofluorescence (IF) studies are needed to determine if dysregulation of regulatory lymphocytes is present in the inflamed sites. Currently, there is a lack of validated anti-feline antibodies that would consistently work well in both cryopreserved and formalin-fixed paraffin-embedded tissue. This shortcoming precluded semiquantitative IHC assessment. Recent immunological discoveries of the significance of T regs and Th17 T cells in immune-mediated disease (30) further underscore the importance of multicolor IF studies. Examination of FCGS affected tissue with IF will allow quantification of specific lymphocyte subsets and aid in our understanding of this disease.

While the majority of cells had intranuclear FOXP3 labeling, occasional sections had intracytoplasmic localization of this epitope. The significance of this in the context of FCGS remains elusive. It was determined by Weed et al. that intracytoplasmic localization of FOXP3 in the CD4+T cells in oral squamous cell carcinoma was associated with cancer recurrence suggesting enhanced immuno-tolerance (31). An association between FCGS and the incidence of squamous cell carcinoma relative to general feline population has not been reported. However, given the association between chronic inflammation and neoplastic transformation and the relative frequency of squamous cell carcinoma invites such studies in cats. Although not validated for cats due to limitation in available antibodies, recent advances in flow cytometric detection of cytoplasmic vs. intranuclear localization of antigens (32) could offer additional insights into the relevance of cytoplasmic localization of FOXP3 in peripheral blood lymphocytes. Collectively, these findings underscore the importance of detailed investigations of lymphocytic subsets in the diseased tissue in patients with immune-mediated oral inflammation as a thorough understanding of the immunology of these conditions may inform therapy.

Transcriptome analysis revealed differential gene expression between healthy and diseased oral tissues (Figure 4, Supplemental Table 1). Hierarchal cluster analysis and principal component analysis point to upregulated gene expression in areas compatible with disease progression (including several inflammation pathways) with a downregulation of genes associated with metabolic pathways and tight junction formation. In agreement with immunohistochemistry, hematology, and immunophenotyping results, genes that were upregulated more than 3–4-fold include genes encoding for cytokines and receptors involved in T and B-cell development, activation, and interaction (IL7R, IL11, MS4A1, CD22, CD37, CD19, IL17). Among listed cytokines, the upregulation of IL17 gene is intriguing because this cytokine has both protective and pathogenetic role in viral infections (33) and is involved in pathogenesis of immune-mediated inflammatory diseases such as inflammatory bowel disease and multiple sclerosis in human patients (30). In humans, IL17 is produced by various immune cells including Th17 lymphocytes, CD8 cells T cells, NK cells, NK T cells, mast cells, and neutrophils. Unfortunately, the investigation of IL17/IL23 axis in cats is limited by the lack of species-specific antibodies.

The KEGG pathway term group genes associated with diseases such as Malaria were enriched more than 5-fold. Most of the genes in this group were associated with lymphocyte adhesion, proliferation, and inflammation. Inflammation genes associated with other pathogens such as Amoebiasis, and Pertussis bacterium were upregulated 3- and 2-fold, respectively. A search for potential sources of antigens beyond the immediate suspects such as Bartonella and Feline herpes virus 1 should be considered. Multiple genes associated with CD8 and NK cell differentiation, activation, adhesion, survival, and apoptosis were upregulated (GZMB, CD8A, ITGAL), indirectly supporting a potential role of intracellular infectious agents in the pathogenesis of FCGS. Although limited to only three FCGS patients, these transcriptome data may serve as a platform for future investigation of the pathogenesis of this disease.

In conclusion, the data from this study highlights the complexity of FCGS in terms of immunology and tissue inflammation. The findings that FCGS is largely a CD8+ disease may inform current and future immunomodulatory interventions. Specifically, it invites further studies evaluating the functional characteristics of CD8+ T cells in FCGS, especially in light of recent reports on association of CD8+ T cell exhaustion and development of immune-mediated disease in humans (34). Also, connecting CD8 T cell function with a reduction in effector CD8 +T cells after MSC therapy may offer important insight on the disruption of injurious immune responses in FCGS (11). In light of recent exciting advances in the cure of FCGS using systemic administration of MSCs, these data may also inform biomarkers predictive of such cures.

## Data Availability Statement

The datasets generated for this study are provided in the Supplemental Table.

## Ethics Statement

The animal study was reviewed and approved by UC DAVIS Institutional Animal Care and Use Committee. Written informed consent was obtained from the owners for the participation of their animals in this study.

## Author Contributions

NV: IHC data acquisition, analysis, interpretation, drafting of the manuscript and final approval of the version to be published. DS: Transcriptome data analysis. BA: Study concept and design, data acquisition and interpretation and drafting of the manuscript. DB: Study design, data interpretation, analysis, drafting of the manuscript and final approval of the version to be published. NT: Data acquisition and analysis, preparation of figures. NW: Data acquisition, interpretation, and analysis, drafting of the manuscript. CG and EB: data acquisition and analysis.

## Conflict of Interest

The authors declare that the research was conducted in the absence of any commercial or financial relationships that could be construed as a potential conflict of interest.

## Supplementary Material

Supplemental Table 1 Raw data of the flow cytometry and transcriptome analyzes.
